# Index and Repeat Ablation for Atrial Fibrillation in Older versus Younger Patients: A Propensity-Score Matching Analysis

**DOI:** 10.14336/AD.2023.0511

**Published:** 2024-02-01

**Authors:** Ana Isabel Molina-Ramos, Amalio Ruiz-Salas, Carmen Medina-Palomo, Víctor Becerra-Muñoz, Jorge Rodríguez-Capitán, Miguel Romero-Cuevas, Ada Carmona-Segovia, Ignacio Fernández-Lozano, Juan José Gómez-Doblas, Manuel Jiménez-Navarro, Francisco Javier Pavón-Morón, Alberto Barrera-Cordero, Javier Alzueta-Rodríguez

**Affiliations:** ^1^Instituto de Investigación Biomédica de Málaga y Plataforma en Nanomedicina (IBIMA Plataforma BIONAND), Universidad de Málaga, 29010 Málaga, Spain.; ^2^Cardiología y Cirugía Cardiovascular-Área del Corazón, Hospital Universitario Virgen de la Victoria, 29010 Málaga, Spain.; ^3^Centro de Investigación Biomédica en Red de Enfermedades Cardiovasculares (CIBERCV), Instituto de Salud Carlos III, 28029 Madrid, Spain.; ^4^Unidad de Arritmias, Hospital Universitario Virgen de la Victoria, 29010 Málaga, Spain.; ^5^Unidad de Arritmias, Servicio de Cardiología, Hospital Universitario Puerta de Hierro, 28222 Majadahonda, Madrid, Spain

**Keywords:** Arrhythmia, atrial fibrillation, catheter ablation, elderly, pulmonary vein reconnection, reablation

## Abstract

Catheter ablation is a well-established rhythm control therapy in atrial fibrillation (AF). Although the prevalence of AF increases dramatically with age, the prognosis and safety profile of index and repeat ablation procedures remain unclear in the older population. The primary endpoint of this study was to assess the arrhythmia recurrence, reablation and complication rates in older patients. Secondary endpoints were the identification of independent predictors of arrhythmia recurrence and reablation, including information on pulmonary vein (PV) reconnection and other atrial foci. Older (*n*=129, ≥70 years) and younger (*n*=129, <70 years) patients were compared using a propensity-score matching analysis based on age, gender, obesity, hypertension, dyslipidemia, diabetes mellitus, dilated left atrium, severe obstructive sleep apnea, cardiac disease, left systolic ventricular function, AF pattern and ablation technique. Arrhythmia recurrence and reablation were evaluated in both groups using a Cox regression analysis in order to identify predictors. During a 30-month follow-up period, there were no significant differences between older and younger patients in the arrhythmia-free survival (65.1% and 59.7%; log-rank test *p*=0.403) and complication (10.1% and 10.9%; *p*>0.999) rates after the index ablation. However, the reablation rate was significantly different (46.7% and 69.2%; *p*<0.05, respectively). In those patients who underwent reablation procedure (redo subgroups), there were no differences in the incidence of PV reconnection (38.1% redo-older and 27.8% redo-younger patients; *p*=0.556). However, the redo-older patients had lower reconnected PVs per patient (*p*<0.01) and lower atrial foci (2.3 and 3.7; *p*<0.01) than the redo-younger patients. A further important finding was that age was not an independent predictor of arrhythmia recurrence or reablation. Our data reveal that the AF index ablation in older patients had a similar efficacy and safety profile to younger patients. Therefore, age alone must not be considered a prognostic factor for AF ablation but the presence of limiting factors such as frailty and multiple comorbidities.

## INTRODUCTION

The population of older adults has been growing in developed countries in recent decades. Atrial fibrillation (AF) is the most frequent arrhythmia in daily practice and its incidence increases with age, which has an important effect in the morbidity and mortality [1, 2].

AF ablation in younger patients has become an established treatment option as implemented in the current guidelines for the treatment of cardiac arrhythmias [3, 4]. However, to date, safety and efficacy data from older patients undergoing catheter ablation for AF are limited and no clinical trial has been conducted specifically in this population. In clinical practice, the adhesion to recommendations in the management of heart rhythm disorders in older patients is obviously influenced by geriatric pathophysiological factors. Moreover, treating older patients with AF remains a significant therapeutic challenge for clinicians because of several reasons. Firstly, older individuals tend to have a larger left atrium (LA) with electrical and structural remodeling, and fibrosis. This can lead to a reduction in the efficacy of pharmacological treatments aimed at maintaining sinus rhythm and may cause significant side-effects [5]. Secondly, the prevalence of both cardiac and non-cardiac comorbidities is typically higher in older patients. For example, the co-occurrence of heart failure is common and may lead to suboptimal adherence to treatment due to anticipated difficulties in managing polypharmacy and the increased risk of drug-drug interactions, which can significantly influence treatment decisions [6]. Thirdly, older individuals with AF are less likely to receive oral anticoagulation therapy. This is primarily because of concerns regarding a higher risk of bleeding associated with oral anticoagulation in this population, despite the evidence supporting the use of these medications [7]. Finally, although the clinical practice guidelines do not postulate on age, the majority of physicians are more conservative in their approach to indicate ablation for AF in older patients. In the last report of the European Heart Rhythm Association (EHRA) about “The clinical management of arrhythmias in elderly patients” with the participation of 50 centers in 20 countries, most participant centers adopted a cautious attitude to AF ablation in this population [8]. Thus, despite only 18.4% of centers applied ablation for AF without age limitation, which was rather low compared with supraventricular tachycardias (89.8%) and ventricular arrhythmias (65.3%), most centers deemed that age would not have impact on the success rate of ablation. Moreover, the published data are still inconsistent in showing that older patients are associated with a higher rate of complications related to ablation procedures.

On the other hand, ageing is an inevitable process and commonly measured by chronological age. A person aged 65 years, or more is often referred to as ‘elderly’ in the published data from the field of Geriatrics [9, 10]. However, the ageing process is not uniform across the population and the chronological age sometimes fails because of differences in genetics, lifestyle and overall health. Despite the fact that older individuals become more heterogeneous with age, elderly is often used as a specific descriptor to describe frail individuals, which is inaccurate and misleading [11]. Furthermore, there is no concrete definition of elderly that appropriately characterize this population and different descriptions for this term have been used in the literature using chronological age (e.g., 65 years or more and 75 years or more) or without any specific measure [12]. Therefore, we have decided to use the term ‘older’ adults as the standard term and a ‘cut-off’ chronological age of 70 years in order to adapt our findings to the usual clinical practice in light of this variability.

Given the background described above, the main purpose of this study was to assess during a 30-month follow-up period the reablation and complication rates after AF catheter ablation in older patients (≥70 years) in comparison with younger patients (<70 years) using a propensity-score matching analysis. Secondary outcomes were the identification of predictors of arrhythmia recurrence and reablation, and the analysis of the pulmonary vein (PV) reconnection and other arrhythmogenic atrial foci observed in the reablation procedure.

## MATERIALS AND METHODS

### Ethics

The study was approved by the Regional Ethics Committee (*Portal de Ética de la Investigación Biomédica de Andalucía-PEIBA*) (study code FA03-1022) in accordance with the Ethical Principles for Medical Research Involving Human Subjects adopted in the Declaration of Helsinki by the World Medical Association (64^th^ WMA General Assembly, Fortaleza, Brazil, October 2013) and Recommendation No. R (97) 5 of the Committee of Ministers to Member States on the Protection of Medical Data (1997), and Spanish data protection act [updated to Regulation (EU) 2016/679 of the European Parliament and of the Council 27 April 2016 on the protection of natural persons with regard to the processing of personal data and on the free movement of such data (General Data Protection Regulation). All patients provided written informed consent for the study protocol and had the opportunity to discuss any questions or issues. All collected data were registered with alphanumeric codes in order to maintain privacy and confidentiality of the participants.

### Study design and participants

This study is derived from a multicenter retrospective cohort study that included a total of 1055 consecutive patients who underwent their first catheter ablation procedure for symptomatic paroxysmal or persistent AF using point-by-point radiofrequency (RF) or single-shot cryoballoon (CB) and were refractory to one or more anti-arrhythmic drugs [13]. The following exclusion criteria were applied: patients under 18 years of age, refusal of informed consent, patients with asymptomatic AF, absence of previous anti-arrhythmic medication, or patients who had undergone a previous ablation procedure. Recruitment was performed at different hospitals in the province of Malaga (Andalusia, Spain) with the coordination of the Hospital Universitario Virgen de la Victoria between January 2009 and January 2019.

From the original cohort, two groups of patients with a first PV isolation for AF were included in the present study based on chronological age and using a propensity score matching analysis: (1) Older group (≥70 years) with 129 patients, 74.0 ± 3.1 years and 55.8% men; and (2) younger group (<70 years) with 129 patients, 56.1 ± 9.6 years and 55.0% men. All participants were monitored during a 30-month follow-up period after the index ablation procedure to detect arrhythmia recurrence and reablation procedure. Specifically, patients who presented with >30s of symptomatic arrhythmia recurrence (i.e., AF, auricular flutter or atrial tachycardia) 3 months after the index ablation (blanking period) during the follow-up period were recommended for a repeat ablation procedure.

Those patients who underwent reablation procedure were divided into the redo-older and redo-younger subgroups, according to the original group of patients. We examined the reconnection of PVs and other atrial foci (non-PV foci) in order to elucidate the precise anatomical region implicated in the arrhythmia recurrence.

### Catheter ablation procedure for AF

The electrophysiological study and catheter ablation procedure for AF were performed under deep sedation without the need for general anesthesia using a combination of midazolam, fentanyl and propofol. The catheter ablation procedure included two main techniques, RF and CB.

The RF ablation was conducted with CARTO® (Biosense Webster, Irvine, CA, USA) and NAVX® (Endocardial Solutions, Saint Paul, MN, USA) guidance systems, with the corresponding open-irrigated ablation catheters and updates throughout the duration of the study: ThermoCool® and ThermoCool SmartTouch® (Biosense, Webster, Irvine, CA, USA); Therapy Cool Path® and Therapy Cool Flex® (Abbot, Chicago, IL, USA). The CB technique was exclusively a single-shot ablation of the PV ostium with 240-ms applications at -40°C using Arctic Front and Arctic Front Advance catheters (Medtronic, Minneapolis, MN, USA). Phrenic stimulation was performed to monitor nerve injury. A CT-scan was previously performed to assess the anatomy of the PVs. For more details see Molina-Ramos *et al*. [13].

### Follow-up after ablation procedure and repeat PV isolation

The total duration of the follow-up was 30 months from the index ablation procedure. Patients were followed up in the outpatient clinic at 3, 6, 12, 18, 24 and 30 months by a cardiologist. At each follow-up time point, patients had an electrocardiogram and a Holter monitor for 24 h or seven days depending on the frequency of symptoms.

In the first follow-up visit at 3 months, ineffective antiarrhythmic therapy was discontinued as routine clinical practice. However, antiarrhythmic treatment was maintained in the case of symptomatic patients or high suspicion of recurrence. All patients received anticoagulant therapy with acenocoumarol or direct oral anticoagulants one month before and 3 months after the index ablation procedure. After that period, the indication for anticoagulant medication was in accordance with the CHA_2_DS_2_-VASc score.

Paroxysmal or persistent episodes of AF, auricular flutter and/or atrial tachycardia continuous for more than 30 seconds after the 3-month blanking period on electrocardiograms or Holter monitor were considered as arrhythmia recurrence and the reablation procedure was performed based on shared decision-making between patient and physician.

All previously isolated PVs were examined during the reablation, and repeat PV isolation was performed in case of reconnected PVs. New linear lesion sets in atria were added (depending on the activation maps obtained in each case) when the PVs were found isolated.

### Statistical Analysis

Baseline sociodemographic and clinical characteristics were presented separately for the older (≥70 years patients) and younger (<70 years patients) groups. Quantitative variables were expressed as mean [(mean ± standard deviation (SD)] or median [median and interquartile range (IQR)]; and statistically analyzed using the Student’s t-test for normal distribution (and the Welch’s correction if necessary) and Wilcoxon rank sum test for non-normal distribution of data. Categorical variables were expressed as frequencies (*n* %) and statistically analyzed using the chi-squared test or Fisher’s exact test.

Older patients were matched with younger patients using a propensity score matching analysis in order to reduce selection bias and confounding variables. The following variables were used to calculate propensity score: age, gender, hypertension, dyslipidemia, diabetes mellitus, dilated LA, obesity, severe obstructive sleep apnea (OSA), cardiac disease, left systolic ventricular function, AF pattern and the ablation technique. For each older patient, a young patient was randomly selected from the pool of candidates according to the nearest neighbor rule (i.e., ± 0.2 SD). Bias reduction and balance between both groups of patients were assessed with standardized differences of covariates.

Recurrence and reablation rates after the index ablation procedure were estimated in the both groups using the Kaplan-Meier method and the survival curves (time-to-event outcomes) were compared using the log-rank test. Cox proportional hazards regression models were used for investigating the association between the endpoints (primary and secondary) and age, as independent factor [14].

All statistical tests were two-sided and the levels of significance was set at 5% (*p*<0.05) Statistical analyses were performed using R software version 4.1.0 (the R Core Team and the R Foundation for Statistical Computing) and SPSS Statistics version 22.0 (IBM Co., Armonk, NY, USA).

**Table 1:** Baseline sociodemographic and clinical characteristics of older and younger patients prior to the index ablation for AF.

	Older group (≥ 70 years) *n* = 129	Younger group (< 70 years) *n* = 129	*p*-value
**Age (years); median (IQR)**	73 (72-76)	58.0 (51-63)	< 0.001^a^
**Sex, male; *n* (%)**	72 (55.8)	71 (55.0)	> 0.999^b^
**BMI (kg/m^2^); mean ± SD**	29.76 ± 4.71	28.95 ± 5.41	0.201^c^
**Obesity (BMI ≥ 30 kg/m^2^); *n* (%)**	26 (20.2)	27 (20.9)	> 0.999^b^
**Active smoking; *n* (%)**	27 (20.9)	37 (28.7)	0.194^b^
**Hypertension; *n* (%)**	94 (72.9)	91 (70.5)	0.782^b^
**Diabetes mellitus; *n* (%)**	26 (20.2)	25 (19.4)	> 0.999^b^
**Dyslipidemia; *n* (%)**	61 (47.3)	64 (49.6)	0.803^b^
**Severe OSA; *n* (%)**	19 (14.7)	19 (14.7)	> 0.999^b^
**LA diameter (mm); mean ± SD**	38.88 ± 5.28	38.76 ± 4.13	0.839^c^
**Dilated LA; *n* (%)**	55 (42.6)	58 (44.9)	0.802^b^
**Non-paroxysmal AF; *n* (%)**	33 (25.6)	34 (26.4)	> 0.999^b^
**First AF onset (months); median (IQR)**	57.5 (18.0-95.5)	51.0 (22.0-102.0)	0.981^a^
**Left systolic ventricular function (%); mean ± SD**	58.56±5.52	58.49±5.35	0.918^c^
**Previous heart disease; *n* (%)** -Dilated cardiomyopathy -Ischemic cardiomyopathy -Hypertensive cardiomyopathy -Hypertrophic cardiomyopathy -Tachymyocardiopathy -Valvular heart disease -Congenital heart disease	35 (27.1) 3 (2.3) 18 (14.0) 8 (6.2) 1 (0.8) 2 (1.6) 2 (1.6) 1 (0.8)	35 (27.1) 3 (2.3) 11 (8.5) 2 (1.6) 2 (1.6) 3 (2.3) 13 (10.1) 1 (0.8)	> 0.999^b^ > 0.999^b^ 0.237^b^ 0.103^b^ > 0.999^b^ > 0.999^b^ 0.006^b^ > 0.999^b^
**Previous revascularization; *n* (%)**	14 (10.9)	9 (7.0)	0.383^b^
**Previous treatment; *n* (%)** -Beta-blockers -Antiarrhythmic drugs -Oral anticoagulant	59 (45.7) 90 (69.8) 104 (80.6)	80 (62.0) 122 (94.6) 87 (67.4)	0.012^b^ < 0.001^b^ 0.023^b^
**CHA_2_DS_2_-VASc score; median (IQR)**	3.00 (2.00-4.00)	2.00 (1.00-2.75)	< 0.001^a^

a Data were analyzed using the Wilcoxon rank sum test

b Data were analyzed using the Fisher’s exact test

c Data were analyzed using the Student’s t test (Welch’s correction) Abbreviations: AF = atrial fibrillation; BMI = body mass index; IQR = interquartile range; LA = left atrium; OSA = obstructive sleep apnea; SD = standard deviation

**Figure 1.**
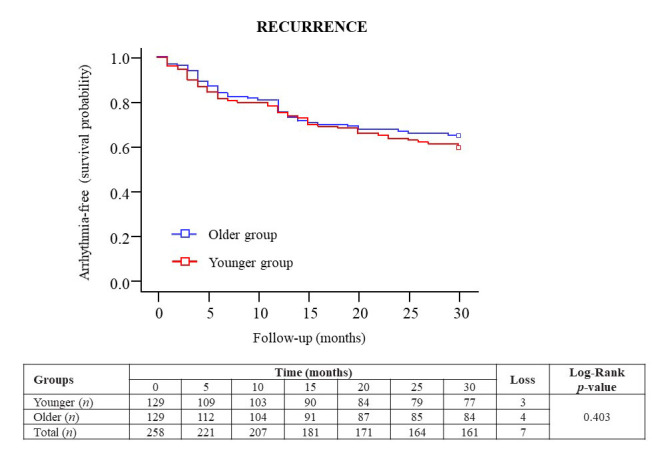
**Kaplan-Meier survival curves of the arrhythmia-free rates during a 30-month follow-up after the index ablation procedure in older (blue) and younger (red) patients with AF**. Abbreviations: AF = atrial fibrillation.

## RESULTS

### Baseline characteristics

Baseline sociodemographic and clinical characteristics of both groups are shown in Table 1.

As expected, there were significant differences in age between both groups (*p*<0.001) and, while older patients had a median age of 73 years, younger patients had a median age of 58 years. Older and younger patients were matched by the ablation AF technique (i.e., RF and CB), cardiovascular risk factors and comorbidities using variables that were previously described as independent predictors of recurrence. Thus, in both groups there were no differences in these variables, such as gender (55%), body mass index (29.4 kg/m^2^) and obesity (20.5%), active smoking (24.8%), hypertension (71.7%), diabetes mellitus (19.8%), dyslipidemia (48.4%) and severe OSA (14.7%). In addition, there were no differences in echocardiographic characteristics, such as LA diameter (38.8 mm), non-paroxysmal AF (26.0%), first AF onset (median: 54.3 months) and left systolic ventricular ejection fraction (58.5 %).

**Table 2:** Identification of predictor variables of arrhythmia recurrence during a 30-month follow-up period in older and younger patients who underwent AF ablation.

Variable	Cox regression model for arrhythmia recurrence		
	HR	95% CI	*p*-value
**Age (younger vs. older)**	0.98	0.48-1.99	0.958
**Sex, male**	0.97	0.55-1.53	0.733
**Obesity (BMI ≥ 30 kg/m^2^)**	1.34	0.76-2.35	0.317
**Active smoking**	1.50	0.82-2.75	0.187
**Hypertension**	1.17	0.68-2.02	0.573
**Diabetes mellitus**	1.18	0.68-2.03	0.555
**Dyslipidemia**	1.02	0.64-1.63	0.928
**Severe OSA**	2.18	1.32-3.61	0.002
**Dilated LA**	1.60	1.06-2.43	0.025
**Non-paroxysmal AF**	1.67	1.09-2.54	0.017
**Early recurrence**	5.82	3.71-9.12	< 0.001

Abbreviations: AF = atrial fibrillation; BMI = body mass index; CI = confidence interval; HR = hazard ratio; LA = left atrium; OSA = obstructive sleep apnea

**Figure 2.**
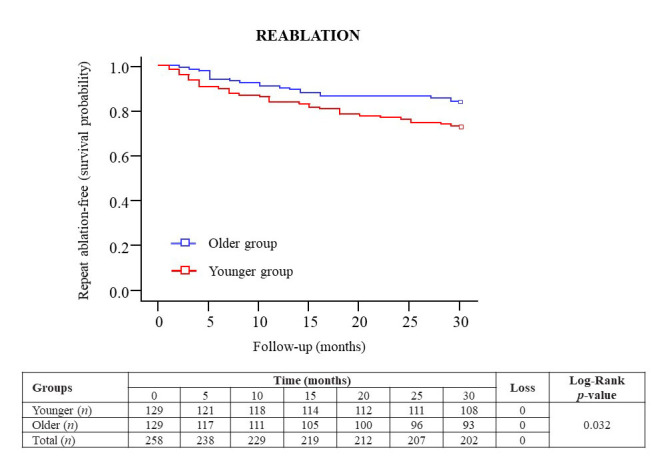
**Kaplan-Meier survival curves of the repeat reablation-free rates during a 30-month follow-up after the index ablation procedure in older (blue) and younger (red) patients with AF**. Abbreviations: AF = atrial fibrillation.

Regarding previous heart diseases, there were no significant differences in the total percentage between both groups (27.1%). However, while the most prevalent heart diseases in older patients were ischemic (14.0%) and hypertensive (6.2%) cardiomyopathies, in younger patients were valvular heart disease (10.1%) and ischemic cardiomyopathy (8.5%). Although there were no differences in the prevalence of previous revascularization (8.9%), we observed differences in previous pharmacological treatments. Specifically, older patients had a significantly lower use of beta-blockers and antiarrhythmic drugs (*p*<0.05 and *p*<0.001, respectively) but a significantly higher use of oral anticoagulants (*p*<0.05) than younger patients.

As expected, we found significant differences in the CHA_2_DS_2_-VASc score between both groups because older patients had a score significantly higher than younger patients (median: 3.0 and 2.0 points, respectively; *p*<0.001).

**Table 3:** Major and minor complications derived from the index ablation in older and younger patients.

Variable	Total *n* = 258	Older group (≥ 70 years) *n* = 129	Younger group (< 70 years) *n* = 129	*p*-value ^a^
**Major complications; *n* (%)**	6 (2.3)	2 (1.6)	4 (3.1)	0.684
**Pericardial tamponade** Atrial-esophageal fistula Stroke Death from the procedure	2 (0.8) 1 (0.4) 1 (0.4) 2 (0.8)	1 (0.8) 0 (0.0) 0 (0.0) 1 (0.8)	1 (0.8) 1 (0.8) 1 (0.8) 1 (0.8)	> 0.999 > 0.999 > 0.999 > 0.999
**Minor complications; *n* (%)**	21 (8.1)	11 (8.5)	10 (7.8)	> 0.999
**Pericardial effusion** Phrenic nerve injury Air embolism Groin-site complications* TIA	3 (1.2) 7 (2.7) 5 (1.9) 4 (1.6) 2 (0.8)	2 (1.6) 5 (3.9) 3 (2.3) 0 (0.0) 1 (0.8)	1 (0.8) 2 (1.6) 2 (1.6) 4 (3.1) 1 (0.8)	> 0.999 0.447 > 0.999 0.122 > 0.999

* Groin-site complications included: vascular pseudoaneurysm, arteriovenous fistula and crural plexopathy.

a Data were analyzed using the Fisher’s exact test Abbreviations: SD = standard deviation; TIA = transient ischemic attack

**Table 4:** Main reasons to refuse repeat ablation procedure in older and younger patients who underwent AF ablation with documented arrhythmia recurrence.

Variable	Older group (≥ 70 years)	Younger group (< 70 years)	*p*-value ^a^
**Arrhythmia recurrence; *n* (total %)**	45 (34.9)	52 (40.3)	0.441
**Refuse reablation; *n* (recurrence %)**	24 (53.3)	16 (30.8)	0.038
**1. Alternative therapeutic approach**	2 (8.3)	1 (6.3)	> 0.999
**2. Symptomatic improvement after the index ablation procedure**	5 (20.8)	8 (50.0)	0.086
**3. Patient-physician agreement in not undergoing reablation**	16 (66.7)	4 (25.0)	0.022
**4. Previous major complications with the index ablation procedure**	1 (4.2)	3 (18.8)	0.283

a Data were analyzed using the Fisher’s exact test Abbreviations: AF = atrial fibrillation

### AF ablation and arrhythmia recurrence

#### Index ablation procedure for AF

During the index ablation, there were no differences in the number of patients treated with RF and CB in both groups (*p*=0.197). Thus, while RF ablation was used in 42 older patients (32.6%) and 53 younger patients (41.0%), CB ablation was used in 87 older patients (67.4%) and 42 younger patients (32.6%), respectively.

The PV isolation with bidirectional block of the cavotricuspid isthmus was successfully performed in 37 older patients (28.7%) and 35 younger patients (27.1%) (*p*=0.890).

#### Arrhythmia recurrence during follow-up

The recurrence after the first ablation is shown in Figure 1 using survival curves of both groups during the follow-up. There were no significant differences in the arrhythmia-free survival curves between older (65.1%) and younger (59.7%) patients (log-Rank test *p*=0.403). Thus, we found no differences between both groups in the number of patients with arrhythmia recurrence and losses at the end of the follow-up period (*p*=0.190).

In the older group, 45 patients (34.9%) experienced recurrence, 28 patients with paroxysmal AF and 17 patients with persistent AF. The primary arrhythmia recurrence was AF in 25 patients, auricular flutter in 18 patients and atrial tachycardia in 2 patients. In the younger group, 52 patients (40.3%) had arrhythmia recurrence, 33 patients with paroxysmal AF and 19 patients with persistent AF. The type of arrhythmia recurrence was AF in 40 patients, auricular flutter in 9 patients and atrial tachycardia in 3 patients. Early recurrence (recurrence detected 3 months after the index ablation) was detected in 19 patients (14.7%) for each group (*p*>0.999).

Antiarrhythmic therapy was maintained in all patients with symptomatic recurrence after ablation procedure. The percentage of electrical cardioversion in the follow-up was significantly higher in the older group (31.0%) than in the younger group (23.3%) (*p*=0.006). However, no differences were observed in the maintenance of the antiarrhythmic drugs at the end of follow-up in both groups (21.7% and 24.8%, respectively) (*p*=0.659).

#### Independent predictors of arrhythmia recurrence

The Cox regression analysis was performed to identify which independent factors were associated with unsuccessful ablation outcomes in terms of arrhythmia recurrence. Table 2 shows that severe OSA [hazard ratio (HR)=2.18 and 95% confidence interval (CI)=1.32-3.61; *p*=0.002], dilated LA (HR=1.60 and 95% CI=1.06-2.43; *p*=0.025), non-paroxysmal AF (HR =1.67 and 95% CI=1.09-2.54; *p*=0.017) and early recurrence (HR=5.82 and 95% CI=3.71-9.12; *p*<0.001) were identified as independent predictors of arrhythmia recurrence. Age was not found to be predictor of recurrence.

### Procedural complications after the index ablation procedure

The Table 3 shows all complications after the single-procedure for AF ablation, which were classified according to the severity. Among major complications, we included cardiac tamponade, atrial-esophageal fistula, stroke and death secondary to ablation. No significant differences in major and minor complication rates were observed between older and younger patients (*p*=0.684 and *p*>0.999, respectively). Unfortunately, one patient from the older group died because of cardiac tamponade and another patient from the younger group died because of atrial-esophageal fistula. It should be noted that PV stenosis was not diagnosed in the sample.

**Table 5:** Identification of predictor variables of repeat ablation procedure during a 30-month follow-up period in older and younger patients who underwent AF ablation.

Variable	Cox regression model for reablation		
	HR	95% CI	*p*-value
**Age (older vs. younger)**	1.49	0.57-3.89	0.421
**Sex, male**	0.88	0.43-1.78	0.724
**Obesity (BMI ≥ 30 kg/m^2^)**	3.60	1.49-8.71	0.004
**Active smoking**	0.92	0.36-2.34	0.084
**Hypertension**	1.32	0.62-2.82	0.474
**Diabetes mellitus**	1.04	0.49-2.24	0.914
**Dyslipidemia**	1.10	0.59-2.05	0.759
**Severe OSA**	2.58	1.32-5.045	0.006
**Dilated LA**	1.89	1.07-3.35	0.029
**Non-paroxysmal AF**	1.31	0.67-2.54	0.422
**Early recurrence**	3.95	2.20-7.08	< 0.001

HR = hazard ratio; LA = left atrium; OSA = obstructive sleep apnea

### Repeat ablation after arrhythmia recurrence

#### Reablation rates during follow-up

The reablation rate is shown in Figure 2 using Kaplan-Meier survival curves in the older and younger groups during the follow-up. There were differences in the repeat ablation-free survival rates between older (83.7%) and younger (72.1%) patients (log-Rank test *p*=0.030). Thus, 21 patients of the older group and 36 patients of the younger group were treated with reablation procedures after the 30-month follow-up, which resulted in significant differences in the reablation rate after existing arrhythmia recurrence (46.7% and 69.2%, respectively; *p*=0.038). In addition, 3 patients of the older group and 13 patients from the younger group were treated with a third ablation procedure (6.7% and 25.0%, respectively; *p*=0.026).

As shown in Table 4, there were several reasons to refuse repeat ablation procedure despite arrhythmia recurrence. In the older group, 24 patients did not receive additional ablation because of the following reasons: (1) two patients (12.5%) underwent atrioventricular node ablation and pacemaker implantation to control heart rate; (2) five patients (20.8%) had symptomatic improvement after the first ablation and decided to continue with oral antiarrhythmic treatment; (3) sixteen patients (66.7%) and the physician agreed not to perform another ablation after assessing the success rate and risk; and (4) one patient (4.2%) reported a major complication and refused their consent to undergo reablation procedure. In the younger group, 16 patients were not treated with a second ablation: (1) One patient (6.3%) underwent reablation after the predefined 30-month follow-up because of late recurrence; (2) eight patients (50.0%) had a symptomatic improvement after the index ablation and decided to continue with oral antiarrhythmic treatment; (3) four patients (25.0%) and the physician agreed not to perform another ablation; and (4) three patients (18.8%) reported major complications related to the index ablation procedure and refused their consent to undergo another ablation procedure. The comparison between both groups for each refusal revealed a significant difference in the number of agreements between the patient and the physician (*p*=0.022).

#### Independent predictors of repeat ablation

Another full-adjusted Cox regression analysis was used to estimate the relative contribution and predictive power of relevant independent variables on the reablation rate in the sample. Table 5 shows that obesity (HR=3.6 and 95% CI=1.49-8.71; *p*=0.004), severe OSA (HR=2.58 and 95% CI=1.32-5.045; *p* =0.006), dilated LA (HR=1.89 and 95% CI=1.07-3.35; *p*=0.029) and early recurrence (HR=3.95 and 95% CI=2.20-7.08; *p*<0.001) were identified as potential predictors of reablation among patients with prior AF ablation. Once again, age was not found to be a predictor of reablation procedure.

### PV reconnection and anatomical distribution of atrial foci in reablation procedures

#### PV reconnection

The presence of PV reconnection (i.e., at least one reconnected PV) in the follow-up was assessed in the 57 procedures of reablation but there were no differences in both redo subgroups [8 patients with PV reconnection in the redo-older subgroup (38.1%) and 10 patients with PV reconnection in the redo-younger subgroup (27.8%); *p*=0.556].

However, there were significant differences in the number of reconnected PVs per patient in the redo-older subgroup compared with the redo-younger subgroup (median: 1 and 2 reconnected PVs, respectively; *p*=0.007). In particular, the number of patients with 0, 1, 2, 3 and 4 reconnected PVs at the time of the reablation procedure was 13 (61.9%), 2 (9.5%), 2 (9.5%), 1 (4.8%) and 3 (14.3%) older patients, respectively; and 10 (27.8%), 8 (22.2%), 8 (22.2%), 4 (11.1%) and 6 (16.7%) younger patients, respectively.

**Table 6:** Exact distribution of the atrial foci in older and younger patients from the redo-subgroups in the repeat ablation procedures.

Anatomical distribution of the 182 atrial foci in the 57 procedures of reablation			
Foci	Redo-older subgroup (≥ 70 years) *n* = 21	Redo-younger subgroup (< 70 years) *n* = 36	*p*-value ^a^
**Total; *n* (rate)**	49 (2.333)	133 (3.694)	0.005
**RSPV; *n* (rate)**	6 (0.286)	15 (0.417)	0.449
**RIPV; *n* (rate)**	7 (0.333)	19 (0.250)	0.570
**LSPV; *n* (rate)**	3 (0.143)	11 (0.306)	0.245
**LIPV; *n* (rate)**	4 (0.191)	14 (0.389)	0.206
**Total PV; *n* (rate)**	20 (0.952)	59 (1.639)	0.031
**LA posterior/roof; *n* (rate)**	14 (0.667)	29 (0.806)	0.572
**LA/RA septum; *n* (rate)**	3 (0.143)	15 (0.417)	0.074
**LA mitral isthmus; *n* (%)**	1 (0.048)	10 (0.278)	0.054
**LAA; *n* (rate)**	0 (0.000)	1 (0.028)	0.445
**RA; *n* (rate)**	2 (0.095)	2 (0.056)	0.614
**SVC; *n* (rate)**	0 (0.000)	1 (0.278)	0.445
**CS; *n* (rate)**	1 (0.048)	6 (0.167)	0.244
**CTI; *n* (rate)**	8 (0.381)	10 (0.278)	0.510
**Total non-PV; *n* (rate)**	29 (1.381)	74 (2.056)	0.065

a Data were analyzed as rates (foci/total *n*) using the chi squared test

Abbreviations: CS = coronary sinus; CTI = cavotricuspid isthmus; LA = left atrium; LAA = left atrial appendage; LIPV = left inferior pulmonary vein; LSPV = left superior pulmonary vein; PA = posterior-anterior; PV = pulmonary vein; RA = right atrium; RIPV = right inferior pulmonary vein; RSPV = right superior pulmonary vein; SVC = superior vena cava

#### Anatomical distribution of atrial foci

**Figure 3.**
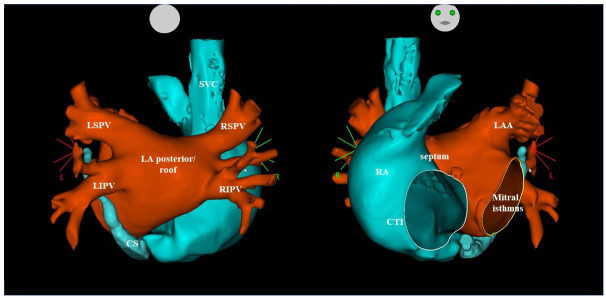
Three-dimensional diagram of the anatomy of the LA and PVs, front (right) and back (left) views.

Multiple atrial foci causing arrhythmia recurrence are shown in Table 6. The anatomical locations of these arrhythmogenic foci are also shown in Figure 3.

There was a total of 182 atrial foci in the 57 reablation procedures, 49 foci (20 PV foci and 20 non-PV foci) in the redo-older subgroup (*n*=21) and 133 foci (59 PV foci and 74 non-PV foci) in the redo-younger (*n*=36) subgroup. The comparison between arrhythmogenic foci rates revealed that redo-older patients had significantly lower total and PV foci rates than redo-younger patients (total foci rates: 2.3 and 3.7, *p*=0.005; and PV foci rates: 1.0 and 1.6, *p*=0.031; respectively). In contrast, there were no significant differences in the non-PV foci rates (*p*=0.065) of both subgroups.

Despite these differences in foci rates, there were no differences between redo-older and redo-younger patients in their anatomical locations and the non-PV foci were more numerous than the PV foci in both subgroups (Table 6). Thus, there were 21 non-PV foci in the redo-older subgroup (59.2%) and 74 non-PV foci in the redo-younger subgroup (55.6%).

Finally, there were no significant differences between paroxysmal and non-paroxysmal AF regarding the presence of non-PV foci [29 patients with paroxysmal AF (76.3%) and 16 patients with non-paroxysmal AF (88.9%); *p*=0.473].

## DISCUSSION

To date, the clinical outcomes of catheter ablation for AF in adults of advanced age are limited, and most of the recommendations are based on extrapolations from younger patient populations. In the present study, we have examined both older (≥70 years) and younger (<70 years) patients with AF and revealed important findings that are summarized as follows: (1) the arrhythmia-free survival rates after a single-procedure for AF ablation were similar in older and younger patients during a 30-month follow-up; (2) there were no differences in major and minor complications derived from the index ablation procedure in older and younger patients; (3) there were significant differences in the repeat ablation-free survival rates between both groups of patients, and the proportion of older patients undergoing repeat ablation procedure was significantly lower than in younger patients (i.e., second ablation in 46.7% and 69.2%, respectively; third ablation in 6.7% and 25.0%, respectively); (4) the incidence of PV reconnection was similar in both redo-older and redo-younger subgroups, but redo-older patients had significantly lower reconnected PVs and arrhythmogenic foci rate than redo-younger patients; and (5) the Cox regression models found that age was not an independent predictor of higher arrhythmia recurrence or reablation during the follow-up period.

Based on the literature, the success rate after the final procedure is 59-87% in patients older than 75 years [15, 16]. However, these studies did not report the effect of AF ablation at the follow-up and, sometimes, the study groups were not comparable according to their baseline characteristics. For this reason, we have designed a study with two groups of patients with different chronological age using a propensity score matching analysis in order to homogenize the sample and reduce selection bias and confounding variables. In addition, we have established a long-term follow-up period of 30 months for all patients. Because the indication for AF ablation in those patients over 70 years of age is relatively low (our current database indicates that only 1 in 10 patients who underwent AF ablation were over 70 years), the recruitment for this study was long and time-consuming.

Patients with AF who underwent a catheter ablation using RF or CB technique were included prospectively from a multicenter cohort of 1055 patients. This cohort of patients is representative of those who are routinely treated in developed countries, mostly hypertensive and overweight-obese patients. In our sample, the time elapsed from the first AF episode to the ablation procedure was high in both age groups, about 4.5 years; in fact, a delay in the index procedure was often observed because the indication for AF ablation was more restrictive for patients who were recruited early. In this respect, several studies have linked the early rhythm-control strategy to an improvement in the prognosis of patients with AF. For example, the EAST-AFNET 4 (Early Treatment of Atrial Fibrillation for Stroke Prevention Trial) study demonstrated that early rhythm-control therapy (i.e., all major antiarrhythmic drugs and AF ablation) is associated with a lower risk of adverse cardiovascular outcomes than usual care in patients older than 75 years with early AF and concomitant cardiovascular conditions [17]. Special mention should be made of the CASTLE-AF (Catheter Ablation versus Standard Conventional Treatment in Patients with Left Ventricular Dysfunction and Atrial Fibrillation) trial, which demonstrated the benefits of AF ablation with prognostic impact in patients with heart failure and a left ventricular ejection fraction of 35% or less. The trial showed a reduction in the composite of death from any cause or hospitalization for worsening heart failure [18]. Because the prevalence of both heart failure and AF increases with age, with rates of more than 10% in those over 70 years old, the beneficial role of catheter ablation in treating both comorbidities, which frequently occur synergistically in older adults, should be highlighted and taken into consideration [19]. Other relevant studies also endorse the early AF ablation to delay the progression to persistent AF [20, 21]. Furthermore, a recent publication has reported that patients with AF can progress to persistent AF while waiting for their catheter ablation appointment (i.e., 10 months waiting list) and suffer from higher rate of AF recurrence after the ablation procedure [22].

Regarding the arrhythmia recurrence, the arrhythmia-free survival curves after the index ablation procedure were similar in older and younger patients (65.1% and 59.7%, respectively) and chronological age was not identified as an independent predictor of recurrence using a Cox proportional hazards regression analysis. This finding is important, given that fewer AF ablation procedures are routinely indicated for this age group in clinical practice because it has been thought that patients of advanced age have a higher risk of recurrence and, therefore, a lower success rate of the procedure. In contrast, other classical risk factors for arrhythmia recurrence, such as the presence of dilated LA, severe OSA, early recurrence and non-paroxysmal AF were identified as independent predictors during a long follow-up; which is in agreement with previous published studies in AF [23-26].

However, although the arrhythmia-free survival rates and complications related to the index procedure were similar in both age groups, the number of patients who underwent repeat ablation during the follow-up period was significantly lower in older patients than in younger patients (46.7% and 69.2%, respectively). Namely, among the 24 older patients who refused to reablation, the main reason in 16 patients (66.7%) was a patient-physician agreement in not undergoing reablation. In contrast, among the 17 younger patients who refused to reablation, only 4 patients (23.5%) had a patient-physician agreement. This difference in reablation procedures reflects a cautious attitude of physicians in not indicating a second procedure in patients of advanced age in the clinical practice. However, we have shown that age was not a predictor of reablation in a Cox proportional-hazards model after adjusting by other independent variables. Furthermore, while the presence of PV reconnection was similar in both age groups in the reablation procedures, redo-older patients had lower reconnected PVs than redo-younger patients, which suggests that the efficacy of isolating the PV ostia in redo-older patients is at least comparable to redo-younger patients. Therefore, chronological age alone should not influence physicians in the choice of catheter ablation for AF but the presence of frailty and comorbidities in these patients, which is more common in adults of advanced age [27, 28].

The ablation strategy focused on the electrical isolation of the PV ostia as initial step, irrespective of whether the patient had paroxysmal, persistent or long-standing persistent AF. Complex fractionated atrial electrograms (CFAE) ablation was only performed in the case of the four PVs remaining isolated in the reablation procedure. This strategy has proven to be effective and safe, as described in the STAR-AF II (Substrate and Trigger Ablation for Reduction of Atrial Fibrillation) clinical trial [29]. Thus, the STAR-AF II trial demonstrated that only PV isolation is not worse than a more extensive ablation procedure (i.e., multiple linear lesions or additional CFAE). Furthermore, a more conservative approach may reduce the incidence of possible peri-interventional complications, while providing comparable arrhythmia-free survival.

Finally, the presence of both PV and non-PV foci as anatomical origin of the arrhythmia recurrence in the reablation procedure was also analyzed. In agreement with the number of reconnected PVs, there were significant differences in the arrhythmogenic foci and redo-older patients had lower PV foci rates than redo-younger patients. However, there were no differences in non-PV foci rates between the redo-older and redo-younger subgroups (75.0% and 85.7%, respectively). In accordance to published data [30], a high rate of non-PV foci was identified in the redo-older subgroup, especially on the posterior and roof of the LA, septum and mitral isthmus. Unlike our study, a previous study by Santangeli *et al.* reported that non-PV foci were most commonly placed in the coronary sinus (54%), left atrial appendage (32%) and superior vena cava and septum (14%). In addition, this study showed that octogenarian patients had significantly higher rate of non-PV foci than non-octogenarian patients (84% and 69%, respectively) [31].

To the best of our knowledge, this is the first study to evaluate the efficacy, safety and repeat ablation rate in older and younger patients with AF using a propensity score matching.

### Limitations

We acknowledge several limitations in our study. First, this is a non-randomized retrospective cohort study that included multivariable analyses with relevant independent variables (covariates) extracted from health records and clinical examinations. However, there may exist other independent and confounding variables that were not accounted for, which may have influenced the study important for the endpoints. Second, this study was conducted with a limited sample size in the southern region of Europe (Málaga, Andalusia, Spain). As such, there are several dietary and environmental factors (e.g., pollution, cultural models of lifestyle and healthcare systems) that can potentially impact the outcomes and introduce biases. Third, the decision to perform a repeat ablation procedure was based on patient and physician preferences, and a substantial number of older patients opted to receive medical treatment instead. Fourth, there has been a continuous improvement in catheter technology over time, including the introduction of contact-force in radiofrequency and the development of new cryoballoon catheters, which may have influenced our results because of the long-term retrospective nature of this study. Despite this limitation, we included consecutive older patients with AF who underwent PV isolation in our clinical practice, and in an effort to homogenize the sample, we used a propensity score-matching analysis to make the results to be compared to younger patients. Finally, our results are based on a patient population with a lower prevalence of heart disease, with less than 1% coexisting tachy-cardiomyopathy and approximately 3% prevalence of dilated or hypertrophic cardiomyopathy. Thus, while our findings may not apply to these particular subgroups, it is noteworthy that our study focused on the most frequently referred patient profile for AF ablation in electrophysiology units. To enhance the external validity of our research, we matched older and younger patients based on the two most frequently used AF ablation techniques (RF vs. CB), and we included patients with both paroxysmal and persistent AF. As a result, our findings are more generalizable to other centers worldwide.

### Conclusions

These findings reveal that older patients with AF who underwent catheter ablation have similar arrhythmia recurrence, PV reconnection and safety profile to younger patients; however, the number of repeat ablation procedures is reduced in this age group compared with younger patients. It is noteworthy that age was not identified as an independent predictor of arrhythmia recurrence or repeat ablation during a 30-month follow-up period, which should be considered by patients and physicians. These data demonstrate that chronological age is not a limiting and vulnerable factor for ablation and reablation procedures in older patients with AF but the existence of frailty and multiple comorbidities, commonly observed in geriatric syndromes. Therefore, older patients with AF need a comprehensive clinical evaluation prior to making a decision about whether ablation procedure is recommended.

## Data Availability

The data presented in this study are available on request from the corresponding author.
